# Exploring miRNA Biomarkers in Major Depressive Disorder: A Molecular Medicine Perspective

**DOI:** 10.3390/cimb46100644

**Published:** 2024-09-27

**Authors:** Cătălin Prodan-Bărbulescu, Laura Andreea Ghenciu, Edward Şeclăman, Georgeta Cristiana Bujor, Virgil Enătescu, Alexandra-Ioana Danila, Ecaterina Dăescu, Luminioara Maria Rosu, Ionuţ Flaviu Faur, Paul Tuţac, Norberth-Istvan Varga, Tanasescu Sonia, Ciprian Duță

**Affiliations:** 1 Faculty of Medicine, “Victor Babeş” University of Medicine and Pharmacy Timisoara, 2nd Eftimie Murgu Square, 300041 Timisoara, Romania; catalin.prodan-barbulescu@umft.ro (C.P.-B.); enatescu.virgil@umft.ro (V.E.); duta.ciprian@umft.ro (C.D.); 2 Department I—Discipline of Anatomy and Embryology, Faculty of Medicine, “Victor Babeş” University of Medicine and Pharmacy Timisoara, 2nd Eftimie Murgu Square, 300041 Timișoara, Romania; alexandra.danila@umft.ro (A.-I.D.); daescu.ecaterina@umft.ro (E.D.); rosu.luminioara@umft.ro (L.M.R.); 3 Department III—Discipline of Physiopathology, Faculty of Medicine, “Victor Babeş” University of Medicine and Pharmacy Timisoara, 2nd Eftimie Murgu Square, 300041 Timișoara, Romania; 4 Department IV—Biochemistry and Pharmacology, Faculty of Medicine, “Victor Babeş” University of Medicine and Pharmacy Timișoara, 2nd Eftimie Murgu Square, 300041 Timișoara, Romania; eseclaman@umft.ro (E.Ş.); bujor.cristiana@umft.ro (G.C.B.); 5 Discipline of Psychiatry, Department of Neurosciences, “Victor Babeş” University of Medicine and Pharmacy Timisoara, 300041 Timișoara, Romania; 6 IInd Surgery Clinic, Timisoara Emergency County Hospital, 300723 Timișoara, Romania; flaviu.faur@umft.ro; 7 Department of General Surgery, “Victor Babeş” University of Medicine and Pharmacy, 300041 Timisoara, Romania; 8 Doctoral School, Department of General Medicine, University of Medicine and Pharmacy Victor Babeş Timisoara, 300041 Timișoara, Romania; paul.tutac@umft.ro (P.T.); norberth.varga@umft.ro (N.-I.V.); 9 Department of Pediatrics, “Victor Babes” University of Medicine and Pharmacy, Eftimie Murgu Sq. No. 2, 300041 Timisoara, Romania; tanasescu.sonia@umft.ro

**Keywords:** major depressive disorder, miRNA, miRNA dysregulation, depression

## Abstract

Major depressive disorder (MDD) is a complex mental health condition with a multifaceted and incompletely elucidated pathophysiology. MicroRNAs (miRNAs) have emerged as potential biomarkers due to their role in gene regulation and the observed dysregulation in MDD. The aim of this study is to detect the presence of specific molecular diagnostic biomarkers in major depressive disorder. This cross-sectional study analyzed plasma miRNA expression in ten MDD patients and eight healthy controls using real-time PCR. Differentially expressed miRNAs were identified using independent *t*-tests, and their diagnostic potential was assessed with ROC curve analysis. Fifteen miRNAs exhibited significant dysregulation in MDD patients. Notably, hsa-miR-29c-3p, hsa-miR-376a-3p, hsa-miR-532-5p, and hsa-miR-339-5p showed excellent discriminatory power (AUC > 0.8). This study identifies differentially expressed plasma miRNAs in MDD, suggesting their potential for improved diagnosis and personalized treatment. However, further validation in larger cohorts and investigation into their functional roles are warranted.

## 1. Introduction

Major depressive disorder (MDD) is a debilitating mental health condition characterized by pervasive sadness, anhedonia (loss of interest or pleasure), and a constellation of associated symptoms, including changes in appetite and sleep patterns, fatigue, and impaired concentration [1]. These symptoms significantly disrupt daily functioning and overall well-being [2]. Despite its challenges, MDD is treatable with interventions such as psychotherapy, pharmacotherapy, or a combination of both, empowering individuals to manage their symptoms and reclaim fulfilling lives [3,4].

Beyond the well-established etiological factors of MDD, such as monoaminergic dysregulation, hypothalamic–pituitary–adrenal axis dysfunction, and genetic predisposition, recent research has focused on the molecular underpinnings of these changes. Of particular interest is the abnormal fluctuation of plasma microRNA (miRNA) levels [3]. MDD’s complex pathophysiology is influenced by an interplay of social, psychological, and genetic factors, with emerging evidence suggesting a role for miRNAs as epigenetic modulators in the central nervous system’s homeostasis and development [1,2,3,4]. Genetic studies indicate a heritability range of 30–40% for MDD, with substantial shared risk factors, underscoring the importance of genetic components in its etiology [5,6,7,8]. The high heritability estimate further supports the potential significance of miRNAs in the disorder’s pathophysiology.

Ribonucleic acid (RNA), the versatile carrier of genetic information, plays a critical role in gene expression and regulation [9]. Among its diverse forms, microRNAs (miRNAs), a class of small non-coding RNAs, have emerged as key regulators of post-transcriptional gene silencing [10]. These short RNA molecules, typically 19–25 nucleotides in length, exert their influence by binding to target messenger RNAs (mRNAs), leading to their degradation or translational repression [11]. Through this intricate mechanism, a single miRNA can fine-tune the expression of multiple genes, significantly impacting a wide range of biological pathways.

Mounting evidence suggests a compelling link between miRNA dysregulation and the pathophysiology of different health conditions, including MDD [12,13,14,15,16]. Studies have reported altered miRNA expression in the brains and peripheral tissues of MDD patients, particularly those involved in regulating neurotransmitters, synaptic plasticity, and stress responses—all crucial factors in MDD’s development and progression [12,17]. Given that approximately 70% of known miRNAs are expressed in the brain and contribute to key neurological processes, their potential as diagnostic biomarkers and therapeutic targets for MDD holds significant promise [17,18,19].

Given the compelling evidence implicating miRNA dysregulation in the pathophysiology of MDD, this study aims to investigate the potential of specific miRNAs as biomarkers for the diagnosis and prognosis of this debilitating disorder.

## 2. Materials and Methods

### 2.1. Study Design and Population

This cross-sectional study included adult patients (age > 18) diagnosed with major depressive disorder (MDD) according to the DSM-IV-TR criteria at the “Eduard Pamfil” Psychiatric Clinic of Timisoara. Data collection took place between 1 September and 1 October 2023. A control group that consisted of age- and sex-matched healthy individuals with no history of psychiatric disorders was also included. All of the participants provided written informed consent prior to enrollment. The study protocol was approved by the Ethics Committee for Scientific Research of the Timisoara County Emergency Hospital (protocol code 136-10.08.2023). Patients who did not provide informed consent were excluded from the study. Data collection also included the age and sex of the participants.

### 2.2. miRNA Extraction and Expression

Venous blood samples were collected from participants into EDTA-coated vacutainers. Plasma was separated via centrifugation and stored at −80 °C until further analysis. miRNA was extracted using the miRNeasy Serum/Plasma kit (Qiagen, Dusseldorf, Germany), and mature miRNA expression was determined using real-time PCR in an ABI 7900HT System (Thermo, Waltham, MA, USA) using the miRCURY LNA miRNA Focus Panels (Qiagen, Dusseldorf, Germany). Real-time PCR data were analyzed using the QIAGEN GeneGlobe Data Analysis Center (Qiagen, Dusseldorf, Germany).

### 2.3. Statistical Analysis

Fold changes in normalized miRNA expression were calculated for all samples, comparing the MDD group to the control group. Fold-Change (2^−ΔCT^) was calculated by dividing the normalized miRNA expression in the MDD group by the normalized expression in the control group.

The normality of the data was assessed visually using histograms. Due to the small sample size, formal statistical tests for normality, such as the Shapiro–Wilk test or the Kolmogorov–Smirnov test, were not performed. An independent *t*-test was employed to assess the statistical significance of differences in miRNA expression between the two groups.

Receiver Operating Characteristic (ROC) curve analysis was subsequently performed on miRNAs, demonstrating statistically significant differences to evaluate their diagnostic potential. The area under the ROC curve (AUC) was calculated for each miRNA, with values closer to 1 indicating higher discriminatory power.

*p*-values less than 0.05 were considered statistically significant.

All of the statistical analyses were conducted using SPSS 19 IBM statistical software (SPSS IBM, New York, NY, USA).

**Figure 1:**
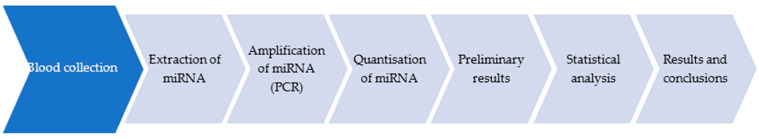
Overview of the trajectory of this study.

Figure 1 describes the methodology used for this study.

## 3. Results

### 3.1. Study Population

The study population consisted of two groups: ten adult patients diagnosed with major depressive disorder (MDD) and eight age- and sex-matched healthy controls with no history of psychiatric disorders. Data collection spanned one month, during which only ten MDD patients provided written informed consent, resulting in the final sample size. Table 1 summarizes the demographic characteristics of the study population.

### 3.2. Identification of MDD-Associated miRNAs

Table 2 presents the specific miRNAs exhibiting significant differential expression in this study. Fifteen hsa-miRNAs demonstrated notable dysregulation in the plasma of patients with major depressive disorder (MDD) compared to healthy controls. The observed dysregulation of these miRNAs suggests their potential as candidate biomarkers for MDD. To facilitate further discussion and analysis, each hsa-miR has been designated with a “BM” prefix (abbreviation from “biomarker”) followed by a numerical identifier (BM1 to BM15), as shown in Table 2. The table also details the fold regulation and corresponding *p*-values for each miRNA.

This study hypothesized a statistically significant difference in the expression levels of 15 selected biomarkers (BM1–BM15) between healthy individuals and those with MDD. To investigate this, an independent *t*-test was employed to compare group means.

The analysis revealed that nine of these miRNAs (BM1, BM5, BM6, BM7, BM9, BM10, BM11, BM12, and BM15) exhibited statistically significant differential expression between the MDD and control groups (*p* < 0.05), suggesting their potential utility as diagnostic markers for MDD. Table 3 details the results of the *t*-tests, including *t*-values and *p*-values, for all 15 miRNAs. The remaining six miRNAs (BM2, BM3, BM4, BM8, BM13, and BM14) did not reach statistical significance and were thus excluded from further analysis.

### 3.3. ROC Curves Analysis

The biomarkers that proved statistical significance in the previous analysis were further evaluated using Receiver Operating Characteristics (ROC) curve analysis to assess their diagnostic accuracy. The area under the ROC curve (AUC) and 95% confidence intervals (CIs) for each miRNA are presented in Table 4.

The analysis identified several biomarkers with AUC values ranging from 0.28 to 0.83, indicating varying degrees of discriminatory power. Those demonstrating sufficiently large AUCs warrant further investigation. Notably, BM1 (hsa-miR-29c-3p), BM9 (hsa-miR-376a-3p), BM10 (hsa-miR-532-5p.), and BM12 (hsa-miR-339-5p.) exhibited AUCs exceeding 0.8, indicating excellent discriminatory ability between MDD patients and healthy controls.

While most miRNAs showed moderate-to-high AUC values, BM5 (hsa-mir-15b-5p) exhibited a notably lower AUC of 0.28, suggesting limited diagnostic utility in differentiating between the two groups.

## 4. Discussion

This study identified fifteen hsa-miRNAs exhibiting significant differential expression in the plasma of individuals with major depressive disorder (MDD) compared to healthy controls. The observed dysregulation of these miRNAs underscores their potential as candidate biomarkers for MDD. Notably, several of these miRNAs have also been implicated in MDD in previous research [20,21], further supporting their relevance in the diagnostic and prognostic landscape of this disorder.

Our findings align with the existing literature regarding the role of specific miRNAs in MDD [22,23]. For instance, members of the let-7 miRNA family, known to be highly expressed in the human brain and crucial for synaptogenesis and neurogenesis, have been associated with MDD in a meta-analysis by Maffioletti et al., which reported the downregulation of hsa-let-7e-5p in MDD patients [24]. In contrast, our study observed the significant upregulation of hsa-miR-532-5p, a finding that diverges from previous reports of its downregulation in MDD [20]. This discrepancy suggests a complex, potentially bidirectional role for this miRNA in MDD, further supported by its involvement in diverse pathologies beyond mental health. The potential of hsa-miR-532-5p as a therapeutic target in MDD has also been suggested by Yan et al. [25], who demonstrated the involvement of this miRNA in stress-related responses and depression. They observed that mice subjected to chronic mild unpredictable stress exhibited reduced hsa-miR-532-5p levels in the hippocampus. Importantly, increasing the expression of hsa-miR-532-5p in these mice led to a decrease in depression-like behaviors and the suppression of inflammatory markers like IL-6, IL-1β, TNF-α, and MCP-1. Nevertheless, the potential of hsa-miR-532-5p as a therapeutic target warrants further exploration.

The significant upregulation of hsa-miR-18b-5p (BM3) and hsa-miR-339-5p (BM12) aligns with previous findings, linking these miRNAs to neuropsychiatric disorders [26,27]. Their roles in glucocorticoid receptor regulation and neuronal subtype-specific expression, respectively, suggest their potential involvement in the disrupted neurotransmission and synaptic plasticity characteristic of MDD.

Interestingly, our study revealed a statistically significant downregulation of hsa-mir-136-3p in MDD patients. This observation suggests a potential negative correlation between hsa-mir-136-3p expression and the presence of depression. Future studies specifically investigating the consistent downregulation of this miRNA in MDD could further solidify its potential as a plasma biomarker with a distinct downregulation pattern in this disorder.

In addition, our ROC curve analysis highlighted two other miRNAs with notable discriminatory power: hsa-miR-29c-3p and hsa-miR-376a-3p. Both exhibited AUC values exceeding 0.8, suggesting excellent potential as diagnostic biomarkers for MDD. Previous studies have linked hsa-miR-29c-3p to various neurological processes, including neuronal differentiation and neuroinflammation [28,29], while hsa-miR-376a-3p has been implicated in stress responses and neurogenesis [30]. The significant upregulation of these miRNAs in MDD patients observed in our study further strengthens their potential relevance in the context of depression and warrants further investigation into their precise roles in MDD pathophysiology.

While this study provides compelling preliminary evidence for the potential of circulating miRNAs as diagnostic biomarkers in MDD, certain limitations warrant consideration. The small sample size (10 MDD patients and 8 controls) limits the generalizability of these findings. Future studies with larger, independent cohorts are necessary to validate these results and ensure their applicability to a broader population. Moreover, the cross-sectional design precludes establishing a causal relationship between the observed miRNA dysregulation and MDD. Longitudinal studies tracking miRNA expression in relation to symptom severity and treatment response would elucidate their temporal and functional roles in disease progression.

Additionally, as inherent to case–control studies, the potential influence of confounding factors, such as medication use, comorbidities, and lifestyle variables, on miRNA expression cannot be entirely discounted. Future research should prioritize rigorous control for these confounders and delve into the mechanistic underpinnings of miRNA dysregulation in MDD, thus deepening our understanding of their role in this complex disorder.

Collectively, these findings suggest that a panel of differentially expressed miRNAs could augment diagnostic accuracy and facilitate personalized treatment monitoring for MDD. While statistical analysis provides a strong foundation for the diagnostic potential of these biomarkers, future research is imperative to validate their clinical utility and establish standardized protocols for their implementation in routine practice.

## 5. Conclusions

This study reveals significant variations in miRNA expression profiles between individuals with major depressive disorder (MDD) and healthy controls. Specifically, we identified fifteen miRNAs with notable dysregulation, several of which exhibited substantial fold regulation changes and increased plasma levels in MDD patients. These findings highlight the potential of these miRNAs as promising diagnostic biomarkers for MDD. While these results offer valuable insights into the molecular underpinnings of MDD, further research is imperative to validate these findings in larger, independent cohorts and to elucidate the precise functional roles of these miRNAs in MDD pathophysiology.

## Figures and Tables

**Table 1:** Demographic characteristics of patients group versus control group.

Variables	Patients with MDD (n = 10)	Healthy Controls (n = 8)
Age in years (median)	43.7	41.75
Male/Female	3/7	2/6

**Table 2:** Specific miRNA package for major depressive disorder.

Number	miRNA ID	Fold Regulation	*p*-Value
BM 1	hsa-mir-29c-3p	3.72	0.017
BM 2	hsa-mir-200a-3p	2.08	0.027
BM 3	hsa-mir-18b-5p	4.28	0.036
BM 4	hsa-mir-335-5p	3.12	0.037
BM 5	hsa-mir-15b-5p	3.38	0.038
BM 6	hsa-mir-320c	3.5	0.039
BM 7	hsa-mir-7-5p	3.32	0.040
BM 8	hsa-mir-532-3p	7.45	0.040
BM 9	hsa-mir-376a-3p	2.45	0.042
BM 10	hsa-mir-532-5p	15.67	0.043
BM 11	hsa-mir-136-3p	−2.22	0.045
BM 12	hsa-mir-339-5p	4.87	0.045
BM 13	hsa-mir-19a-3p	2.74	0.045
BM 14	hsa-mir-33a-5p	2.68	0.047
BM 15	hsa-mir-483-5p	3.84	0.048

**Table 3:** Application of the independent *t*-test.

BM/miRNA	*t*	*p*-Value	
		One-Sided *p*	Two-Sided *p*
BM1 hsa-mir-29c-3p	2.612	0.009	0.019
BM2 hsa-mir-200a-3p	0.724	0.240	0.479
BM3 hsa-mir-18b-5	0.947	0.179	0.358
BM4 hsa-mir-335-5p	0.844	0.205	0.411
BM5 hsa-mir-15b-5p	1.726	0.035	0.071
BM6 hsa-mir-320c	1.936	0.042	0.084
BM7 hsa-mir-7-5p	2.560	0.010	0.021
BM8 hsa-mir-532-3p	1.276	0.110	0.220
BM9 hsa-mir-376a-3p	2.579	0.010	0.020
BM10 hsa-mir-532-5p	2.242	0.020	0.040
BM11 hsa-mir-136-3p	−2.520	0.011	0.023
BM12 hsa-mir-339-5p	3.225	0.003	0.005
BM 13 hsa-mir-19a-3p	1.099	0.144	0.288
BM 14 hsa-mir-33a-5p	1.594	0.065	0.131
BM15 hsa-mir-483-5p	2.107	0.026	0.051

**Table 4:** Area under the Receiver Operating Curve for biomarkers.

Marker	Area ± Std. Error	95% CI
BM1	0.83 ± 0.10	(0.64–1.03)
BM5	0.28 ± 0.12	(0.04–0.53)
BM6	0.72 ± 0.13	(0.50–1.00)
BM7	0.73 ± 0.13	(0.48–0.98)
BM9	0.81 ± 0.10	(0.61–1.00)
BM10	0.83 ± 0.09	(0.64–1.00)
BM11	0.78 ± 0.11	(0.56–1.00)
BM12	0.83 ± 0.09	(0.64–1.00)
BM15	0.75 ± 0.13	(0.50–1.00)

## Data Availability

Data are contained within this article.
